# *Trametes versicolor* Laccase-Based Magnetic Inorganic-Protein Hybrid Nanobiocatalyst for Efficient Decolorization of Dyes in the Presence of Inhibitors

**DOI:** 10.3390/ma17081790

**Published:** 2024-04-13

**Authors:** Sanjay K. S. Patel, Rahul K. Gupta, Karthikeyan K. Karuppanan, Deepak K. Padhi, Sampathkumar Ranganathan, Parasuraman Paramanantham, Jung-Kul Lee

**Affiliations:** Department of Chemical Engineering, Konkuk University, Seoul 05029, Republic of Korea; sanjaykspatel@gmail.com (S.K.S.P.); guptarahul9m@gmail.com (R.K.G.); karthikk1529@gmail.com (K.K.K.); dkpadhi7@gmail.com (D.K.P.); sampathbiotech@gmail.com (S.R.); parasupondiuni@gmail.com (P.P.)

**Keywords:** acute toxicity, magnetic nanoparticles, protein-inorganic hybrid, stability, *Trametes versicolor* laccase, synthetic dye

## Abstract

In the present investigation, an ecofriendly magnetic inorganic-protein hybrid system-based enzyme immobilization was developed using partially purified laccase from *Trametes versicolor* (*Tv*Lac), Fe_3_O_4_ nanoparticles, and manganese (Mn), and was successfully applied for synthetic dye decolorization in the presence of enzyme inhibitors. After the partial purification of crude *Tv*Lac, the specific enzyme activity reached 212 U∙mg total protein^−1^. The synthesized Fe_3_O_4_/Mn_3_(PO_4_)_2_-laccase (Fe_3_O_4_/Mn-*Tv*Lac) and Mn_3_(PO_4_)_2_-laccase (Mn-*Tv*Lac) nanoflowers (NFs) exhibited encapsulation yields of 85.5% and 90.3%, respectively, with relative activities of 245% and 260%, respectively, compared with those of free *Tv*Lac. One-pot synthesized Fe_3_O_4_/Mn-*Tv*Lac exhibited significant improvements in catalytic properties and stability compared to those of the free enzyme. Fe_3_O_4_/Mn-*Tv*Lac retained a significantly higher residual activity of 96.8% over that of Mn-*Tv*Lac (47.1%) after 10 reuse cycles. The NFs showed potential for the efficient decolorization of synthetic dyes in the presence of enzyme inhibitors. For up to five reuse cycles, Fe_3_O_4_/Mn-*Tv*Lac retained a decolorization potential of 81.1% and 86.3% for Coomassie Brilliant Blue R-250 and xylene cyanol, respectively. The synthesized Fe_3_O_4_/Mn-*Tv*Lac showed a lower acute toxicity towards *Vibrio fischeri* than pure Fe_3_O_4_ nanoparticles did. This is the first report of the one-pot synthesis of biofriendly magnetic protein-inorganic hybrids using partially purified *Tv*Lac and Mn.

## 1. Introduction

Enzymes, as natural catalysts, have been utilized by humans for centuries in various applications, such as in the production of cheese, brewing processes, baking, and the production of wines and alcohol [1,2]. They are essential for numerous biocatalytic industrial processes, including biofuel and food production, and pharmaceutical manufacturing [3,4,5]. However, the traditional use of enzymes in these processes, whether free or immobilized enzymes, has limitations [6,7]. For example, free enzymes are often unstable and can be deactivated or degraded easily under harsh reaction conditions. Additionally, free enzymes are difficult to recover and reuse, leading to increased production costs [8,9]. To address these limitations, researchers have explored the immobilization of enzymes, which involves immobilizing to a support. This approach offers several potential advantages, including improved enzyme stability, enhanced catalytic efficiency, and easier separation and recovery of enzymes [10,11]. Despite these advantages, there remains scientific gaps and limitations in enzyme immobilization that warrant further research. One limitation is the potential loss of enzyme activity during the immobilization process. This loss of activity can be caused by various factors, such as conformational changes, reduced accessibility to substrates, or limited diffusion of substrates and products within the immobilization support [11,12]. Other limitations include the potential for enzyme inactivation due to interactions with the immobilization support or other nearby enzymes, as well as difficulties in achieving optimal enzyme loading. Further research is required to better understand these limitations and develop strategies to overcome them [11,13,14].

Advancements in technology have led to the development of novel methods for enzyme immobilization, including the use of inorganic-protein hybrid systems that are known as nanoflowers (NFs) because their morphology resembles that of flowers [15,16]. In an established mechanism, NF synthesis occurs in phosphate-buffered saline (PBS) through three steps: (i) nucleation via interaction of metal ions with a nitrogen group available in the protein, (ii) followed by aggregation, and (iii) anisotropic growth [17]. Metals such as cobalt (Co), copper (Cu), manganese (Mn), and zinc (Zn) have been widely demonstrated for the immobilization of enzymes as NFs [11,16]. These systems combine the unique properties of inorganic materials with the catalytic activity of enzymes, resulting in enhanced enzyme stability and effectiveness. Moreover, these inorganic-protein hybrids have been utilized for enzyme immobilization, which allows enzymes to be securely attached to a solid support. Initially, the metal ions form complexes with the amide groups of the protein molecules and functional activated nanoparticles. Subsequently, the nanoparticles and metal/phosphate nuclei continue to grow as the enzyme integrates into the hybrid assemblies. Finally, larger nanoparticles and metal-based enzyme hybrid NFs form after longer incubation periods [11,17]. This immobilization technique improves the efficiency and reusability of enzymes in various applications, such as pharmaceutical production and green synthesis processes [16,18]. The combination of genetic engineering techniques and the utilization of inorganic-protein hybrid systems for enzyme immobilization offers promising solutions to overcome the drawbacks associated with enzyme instability and make enzymes more suitable for industrial applications [12,19,20]. One promising approach for enzyme immobilization is the use of magnetic inorganic-protein hybrids, which offer several advantages. These hybrids combine the magnetic properties of inorganic materials with the functional properties of proteins, such as enzymes [21,22]. This allows for the efficient and convenient recovery of the immobilized enzyme using magnetic separation techniques [11].

Laccase plays a crucial role in dye decolorization. It is an enzyme widely used in various industries, including textile and dyestuff manufacturing, because of its ability to oxidize aromatic amines and degrade azo dyes via a nonspecific, free radical-mediated mechanism [19,23]. Moreover, laccase can be produced from various sources, including fungi (*Trametes versicolor*), plants, and bacteria. Therefore, they provide diverse options for industrial applications [24,25]. The use of laccase for dye decolorization has several advantages. These include its effectiveness in removing dyes by forming relatively harmless byproducts [26,27]. Mechanistically, laccases act through direct phenolic substrate oxidation and indirect oxidation of non-phenolic substrates in the presence of a natural/synthetic mediator with a high redox potential, or through coupling reactions via reactive intermediate radicals created in a direct oxidation process [28,29]. Additionally, laccase is more stable and versatile than other enzymes because it can function under a wide range of temperature and pH conditions. Furthermore, laccase functions efficiently in both aerobic and anaerobic environments, making it suitable for various biological treatment systems [30,31,32]. Overall, laccase is a valuable enzyme for dye decolorization because of its effectiveness, versatility, and environmentally friendly nature. Therefore, the immobilization of laccase onto magnetic inorganic-protein hybrids as NFs enhances the stability and reusability of the enzyme and provides a larger surface area for substrate binding, resulting in increased catalytic activity [11,19]. Few reports have been published on magnetic inorganic-protein hybrid-based NFs [20,33]. Mechanistically, magnetic inorganic-protein hybrid NFs are synthesized via the interaction of 3-aminopropyltriethoxysilane (APTES)-functionalized magnetic (Fe_3_O_4_) nanoparticles with the enzyme, followed by precipitation in the presence of desirable metal ions [11,33]. In this study, partially purified laccase from *T. versicolor* (*Tv*Lac), functionally activated Fe_3_O_4_ nanoparticles, and Mn-based synthesis of the magnetic inorganic-protein hybrid NFs, Fe_3_O_4_/Mn_3_(PO_4_)_2_-laccase (Fe_3_O_4_/Mn-*Tv*Lac) and Mn_3_(PO_4_)_2_-laccase (Mn-*Tv*Lac) NFs, were evaluated to improve the properties of *Tv*Lac. After immobilization, significant improvements in the *Tv*Lac properties were observed, including its catalytic activity, pH and temperature profiles, stability, and reusability. Furthermore, these hybrid NFs were successfully applied in the decolorization of dyes in the presence of enzyme inhibitors.

## 2. Materials and Methods

### 2.1. Materials

Magnetic nanoparticles (Fe_3_O_4_) were bought from Nanostructured and Amorphous Materials, Inc. (Houston, TX, USA). Ultrapure-water and PBS were obtained from Life Technologies, Carlsbad, CA, USA. 2,2-azino-bis(3-ethylbenzothiazoline-6-sulfonate) (ABTS), Cu sulfate (CuSO_4_), Mn sulfate (MnSO_4_), APTES, potato dextrose (PD) broth (PDB), dithiothreitol, fluorescein isothiocyanate (FITC), and l-cysteine were acquired from Sigma-Aldrich (St. Louis, MO, USA). Coomassie Brilliant Blue R-250 (CBBR-250) and xylene cyanol were obtained from BioShop (Canada Inc., Burlington, ON, Canada). Other analytic reagents were purchased from commercial suppliers.

### 2.2. Laccase Production and Partial Purification

For laccase production, mycelia of *T. versicolor* (~five disks with a 5 mm diameter) grown on a PD agar (five days) were inoculated into 100 mL PDB (500 mL conical flask) for incubation for five days (30 °C) with slight agitation of 180 rpm. Moreover, 10% (*v*/*v*) precultures were inoculated into 1 L of basal medium (pH 5.0) containing CuSO_4_ (0.1–0.5 mM) as a laccase inducer [2]. The *Tv*Lac production profile was analyzed by measuring enzyme activity for up to eight days of incubation. The supernatant was retrieved by centrifugation (6000 rpm, 30 min, and at 4 °C) and filtered by a membrane (0.22 µm, Millipore, Bedford, MA, USA). Further, it was purified via ultrafiltration using a 30 kDa cut-off membrane (Viva-flow; Vivascience, Hannover, Germany). Total protein content was measured using the Bradford method (4:1 ratio of supernatant to Bradford reagent) at 595 nm with bovine serum albumin as the protein standard [34]. For further use, partially purified laccase was kept at 4 °C [2].

### 2.3. Magnetic Nanoparticles and Laccase-Based Protein-Inorganic Hybrid Nanoflowers Synthesis

Initially, Fe_3_O_4_ was activated using APTES, as previously described [23]. The synthesis of magnetic nanoparticle-based protein-inorganic hybrids as Fe_3_O_4_/Mn-*Tv*Lac NFs was accomplished in 50 mL of 100 mM PBS (pH 6.0), including APTES-activated Fe*_3_*O_4_ (5 mg), partially purified laccase (0.25 mg∙mL^−1^), and 0.5 mL MnSO_4_ (200 mM) at 4 °C for incubation up to 24 h under mild shaking (60 rpm). Similarly, the synthesis of Mn-*Tv*Lac was achieved without Fe_3_O_4_ nanoparticles under similar conditions. The residual protein concentration in the supernatant was measured using the Bradford method after separation, as previously reported [34,35]. An encapsulation yield (EY) was determined by 100 × ratio of the immobilized amount of enzyme to their initially added amount. The RA of NFs was measured as follows (Equation (1)):

RA (%) = total specific activity of immobilized laccase/initial specific activity of free laccase × 100(1)

### 2.4. Laccase Activity Measurements

Enzyme activity was assessed by spectrophotometer at 420 nm using 1 mM of ABTS (ε_max_ = 3.6 × 10^4^∙M^−1^∙cm^−1^) as a substrate [2]. The activity was defined in international units, which denotes the amount of enzyme required to form 1 mol of product per min using ABTS in buffer solution (pH, 3.5) at 25 °C.

### 2.5. Characterization of Immobilized Laccase as Nanoflowers

Using ABTS, the free *Tv*Lac, Mn-*Tv*Lac, and Fe_3_O_4_/Mn-*Tv*Lac NFs activities were evaluated at various pH (2.5–6.0). The influence of temperature on enzyme activity was assayed over the range of 25–85 °C at the optimum pH values under assay conditions. Nonlinear regression (GraphPad Prism 5.0; GraphPad Software, La Jolla, CA, USA) measurements were acquired to determine kinetic parameters using 0.005–2.0 mM of ABTS at 25 °C.

### 2.6. Stability, Reusability, and Leaching Measurement

Initially, room temperature (25 °C) stability of free *Tv*Lac, Mn-*Tv*Lac, and Fe_3_O_4_/Mn-*Tv*Lac NFs was investigated by assessing the residual activity at different intervals in sodium-citrate buffer (100 mM, at optimum pH values) up to 72 h of incubation. Similarly, storage stability (at 4 °C) was assessed for 30 days of incubation. Thermostability was determined between temperatures of 40–80 °C. The initial activity (residual) was considered 100%. The reusability of Mn-*Tv*Lac and Fe_3_O_4_/Mn-*Tv*Lac was determined under standard assay conditions for 10 reuse cycles. The immobilized laccase was recovered through centrifugation (1000 rpm, 10 min, and at 4 °C), pursued by buffer washing (two times), and subsequently employed in the next cycle. Leaching of the immobilized laccase was estimated by assessing the supernatant protein (total) to the initial immobilized enzyme ratio ×100 [2].

### 2.7. Acute Toxicity Analysis

The toxicity of the synthesized NFs was measured towards *Vibrio fischeri* using a Microtox, Model 500 Analyzer (Modern Water, London, UK). In brief, different dilutions of NFs (heat-inactivated, 2.0 mg∙mL^−1^) and pure Fe_3_O_4_ nanoparticles (1.0 mg∙mL^−1^) were prepared according to the manufacturer’s standard protocol, and a decline in the luminescence intensity of *V. fischeri* was monitored for 30 min. Effective concentration (EC) values of the synthesized NFs were denoted as EC_50_, where a 50% reduction in the luminescence of the bacteria was observed after 30 min of incubation.

### 2.8. Decolorization of Synthetic Dyes in the Presence of Laccase Inhibitors

The decolorization of the synthetic dyes, CBBR-250 (λ_max_ = 585 nm) and xylene cyanol (λ_max_ = 615 nm) was assessed in the presence of laccase inhibitors (0.1 mM), such as dithiothreitol, Fe(II), and l-cysteine. In brief, a 10 mL reaction mixture was added to a 50 mL conical flask containing dyes (120 µg∙mL^−1^), inhibitor, and free *Tv*Lac or NFs in buffer (50 mM) under optimum pH conditions at 25 °C for incubation up to 72 h with shaking (100 rpm). Repeated batch decolorization of the dyes was also measured for 12 h of incubation in the presence of Fe^2+^ (0.1 mM) for up to five reuse cycles. NFs were collected through centrifugation at 1000 rpm for 10 min (4 °C) and subsequently applied to the next cycle. The decolorization efficiency at zero cycles was considered 100%. The decolorization percentage was calculated as follows (Equation (2)) [36]:

Decolorization (%) = absorbance after incubation/absorbance at the initial point × 100 (2)

### 2.9. Instrumental Measurements and Statistical Analysis

The field-emission scanning electron microscopy (SEM) measurements of the synthesized NFs were achieved using a JSM-6700F, JEOL, Tokyo, Japan [35]. Diffraction patterns were assessed via X-ray diffraction (XRD, X’pert PRO MPD X-ray, Malvern Panalytical, Malvern, UK). All absorption analysis was achieved by UV–Vis spectrophotometer (Jenway Scientific 6705, Staffordshire, UK) [37]. FITC-labeled enzymes were examined by confocal laser scanning microscopy (CLSM, FV1000 confocal microscope, Olympus, Center Valley, PA, USA) [35]. The magnetic properties of the Fe_3_O_4_ nanoparticles and NFs were analyzed at 298 K using a SQUID/VSM magnetometer, MPMSR3, San Diego, CA, USA. Experimental data values were presented as mean ± standard deviations, and statistical significance was investigated via ANOVA (α = 0.05; GraphPad Prism 5).

## 3. Results and Discussion

### 3.1. TvLac Production and Purification

**Figure 1:**
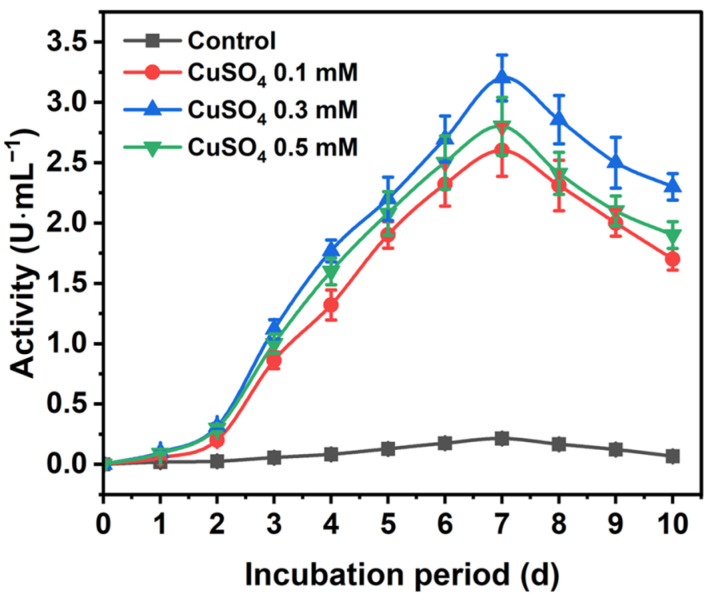
*Trametes versicolor* laccase production profile in the presence of CuSO_4_ as an inducer.

The *Tv*Lac production profile using various inducers is presented in Figure S1. Among different laccase mediators, Cu showed higher residual activity. Initially, laccase activity elevated with increasing incubation for up to seven days, with a crude activity reaching 3.2 U∙mL^−1^ (total activity of 3200 U∙L^−1^) (Figure 1 and Figure S2). Subsequently, the activity significantly declined to 2.3 U∙mL^−1^ after 10 days of incubation. Here, a 15-fold increase in *Tv*Lac production using CuSO_4_ (0.3 mM) was observed compared to the control. Furthermore, *Tv*Lac obtained from a seven-day culture was partially purified via ultrafiltration using a 30 kDa cut-off spin column (Corning, AZ, USA). The specific activity of the partially purified *Tv*Lac increased to 212 U∙mg total protein^−1^ with a fold purification and yield of 1.5 and 66.7%, respectively (Table S1). Previously, *Coriolopsis gallica* showed a low crude laccase production of 31 U∙mg protein^−1^ using inducers [38].

### 3.2. Immobilization of TvLac Using Magnetic Nanoparticles and MnSO_4_ through Protein-Inorganic Hybrid Nanoflowers

The immobilization of partially purified *Tv*Lac as protein-inorganic hybrid NFs was performed using APTES-activated Fe_3_O_4_ nanoparticles and MnSO_4_ in PBS for 24 h of incubation at 4 °C. The synthesized Fe_3_O_4_/Mn-*Tv*Lac NFs exhibited EY and RA values of 85.5% and 245%, respectively (Table 1). A slightly higher EY of 90.3% and RA of 260% were observed for NFs synthesized without Fe_3_O_4_ nanoparticles (Mn-*Tv*Lac). Under assay conditions, free *Tv*Lac showed an RA of 127% in the presence of Mn ions (2 mM) (Table S2). Previously, purified *Tv*Lac immobilized using Co-based NFs, such as laccase@Co_3_(PO_4_)_2_·H NFs, showed a significantly reduced RA of 60.9% under a similar incubation period of 24 h [39]. In contrast, at a longer incubation time of 72 h, laccase NFs synthesized using Ni as laccase@Ni_3_(PO_4_)_2_·H NFs exhibited a low RA of 75% [40]. These results suggest that the immobilization of *Tv*Lac as NFs using magnetic nanoparticles and Mn is more effective for retaining a higher RA.

**Figure 2:**
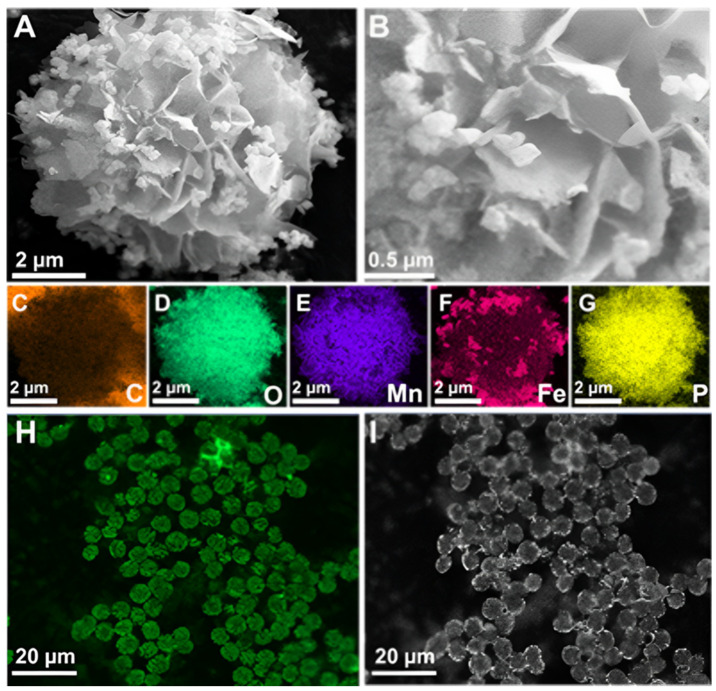
Field-emission scanning electron microscopy examination of (**A**,**B**) magnetic inorganic-protein hybrids as Fe_3_O_4_/Mn-*Tv*Lac, (**C**–**G**) elemental mapping analysis for carbon (**C**), oxygen (**D**), manganese (**E**), iron (**F**), and phosphorus (**G**), and (**H**,**I**) confocal laser scanning microscope images of the synthesized *Tv*Lac hybrid NFs labeled with FITC under green channel (**H**) and bright-field (**I**).

Synthesis incubation conditions and laccase origin highly influence the size and structural morphology of synthesized inorganic-protein hybrid NFs [39,40]. SEM was used to analyze the morphological structures of the synthesized inorganic-protein hybrids. The size of NFs increased from 0.7 µm at 2 h to 7 µm at 24 h of incubation at 4 °C (Figure S3). Under these conditions, the EY rose from 46.1% to 85.5%, with an optimum RA of 245% at 24 h (Table S3). The synthesized Fe_3_O_4_/Mn-*Tv*Lac and Mn-*Tv*Lac NFs showed a flower-like morphology with an average size of 7–9 µm (Figure 2A,B and Figure S4). Under a similar incubation conditions (24 h at 4 °C), the flower-like morphology of Cu-based laccase NFs exhibited higher sizes of ~50 µm [41]. NFs based on Co and laccase showed a size of nearly 2 µm with architecturally altered flower-resembling aggregates [39]. In another study, Ni-based laccase NFs exhibited hierarchically structured hybrids with sizes up to 10 µm [40]. In contrast, Zn-based *Tv*Lac NFs showed a larger size of 25 µm with a flake-like morphology [42]. Elemental mapping was performed to confirm the composition of the synthesized Fe_3_O_4_/Mn-*Tv*Lac NFs. The presence of carbon, Fe, Mn, and phosphorus (P) suggests the successful formation of magnetic inorganic-protein hybrid NFs (Figure 2C–G). Similarly, the presence of Mn, nitrogen, and P confirms Mn-*Tv*Lac NF formation (Figure S4). To validate *Tv*Lac immobilization, FITC-labeled *Tv*Lac was used to synthesize NFs under similar immobilization conditions (Figure 2H,I). In the CLSM analysis, the visualization of green fluorescence in the synthesized magnetic NFs confirmed *Tv*Lac immobilization. The synthesized hybrid NFs XRD patterns are shown in Figure S5. The relative peak locations of Fe_3_O_4_/Mn-*Tv*Lac and Mn-*Tv*Lac NFs were identical to Mn_3_(PO_4_)_2_·3H_2_O (JCPDS No. 03–0426) XRD patterns, Fe_3_O_4_ (JCPDS No. 75–0449), and protein-Mn_3_(PO_4_)_2_·3H_2_O hybrids, validating the synthesis of Mn-based magnetic inorganic-protein hybrids [43,44]. The Fe_3_O_4_ nanoparticles exhibited 24.2 electromagnetic unit∙g^−1^ (emu∙g^−1^) of magnetization saturation, while Fe_3_O_4_/Mn-*Tv*Lac showed 15.0 emu∙g^−1^ (Figure S6A). This finding confirms that the synthesized hybrids retained their magnetic properties, which can be quickly recovered by applying an external magnetic field (Figure S6B,C) [11,21].

### 3.3. Characterization of Free and Immobilized TvLac as Nanoflowers

**Figure 3:**
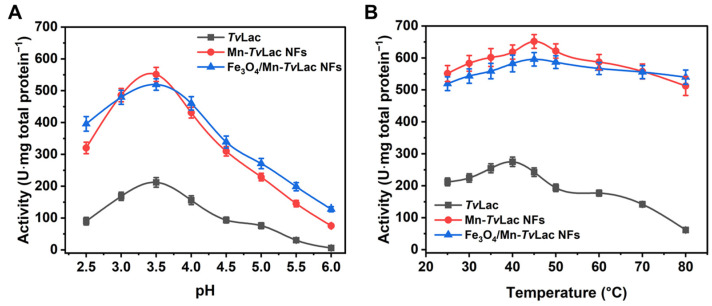
Activity profiles of free and immobilized *Tv*Lac at different (**A**) pH values and (**B**) temperatures.

The free and immobilized *Tv*Lac pH profiles were assessed over a range of 2.5–6.0 in a buffer solution. Free *Tv*Lac had an optimum pH of 3.5 (Figure 3A). After being immobilized as magnetic NFs, a shift in the optimum pH from 3.5 to 4.0 was observed for immobilized *Tv*Lac as Fe_3_O_4_/Mn-*Tv*Lac and Mn-*Tv*Lac NFs. In contrast, no change in the optimal pH was recorded for free laccase and its NFs based on Co and Cu [39,41]. The Fe_3_O_4_/Mn-*Tv*Lac and Mn-*Tv*Lac NFs showed better activity profiles than those of free *Tv*Lac at lower and higher pH values of 2.5 and 6.0, respectively, under optimal conditions. Under these pH conditions, Fe_3_O_4_/Mn-*Tv*Lac and Mn-*Tv*Lac NFs showed up to 1.9- and 14.2-fold higher residual activity than the *Tv*Lac with values of 42.5% at pH 2.5 and 2.8% at pH 6.0, respectively. Previously, similar pH profiles were reported for free and Cu-NFs-based laccase [41]. A similar shift in the temperature-activity profiles of the Fe_3_O_4_/Mn-*Tv*Lac and Mn-*Tv*Lac NFs was observed over that of free *Tv*Lac (Figure 3B). Optimal temperature values of 40 °C for free *Tv*Lac and 45 °C to immobilized *Tv*Lac as Fe_3_O_4_/Mn-*Tv*Lac and Mn-*Tv*Lac NFs were measured. In contrast, a similar optimum temperature value was noted for free and immobilized laccase NFs synthesized using Cu and Co ions [41,45]. Another study on laccase immobilization using metal-organic frameworks showed analogous optimal temperature values for free and encapsulated enzymes [46]. Further, an increase in temperature above the optimum values resulted in the continual decline of the residual activity of *Tv*Lac up to 80 °C. At 80 °C, the *Tv*Lac, Fe_3_O_4_/Mn-*Tv*Lac, and Mn-*Tv*Lac NFs retained residual activities of 22.4%, 78.6%, and 90.6%, respectively. The Mn-based NFs displayed a wide pH and temperature activity profiles compared to the corresponding free *Tv*Lac. Above results imply that encapsulating partially purified *Tv*Lac through magnetic nanoparticles and Mn-based inorganic-protein hybrids could be more advantageous for shifting pH and temperature optimal values than when immobilizing purified-laccase using Co- or Cu-based NFs [39,41,45].

Among various substrates, *Tv*Lac showed high substrate specificity towards ABTS (Table S4). Using ABTS as the substrate, the kinetic parameter values of partially purified *Tv*Lac were observed, with a *K*_m_ of 38.1 µM and *V*_max_ of 219 µmol∙min^−1^∙mg protein^−1^ (Table 2). Fe_3_O_4_/Mn-*Tv*Lac and Mn-TvLac NFs exhibited adequate affinity against ABTS compared to the free *Tv*Lac, indicating a decline in *K*_m_ with corresponding values of 17.6 and 17.1 µM, respectively. Previously, the comparable substrate affinity (*K*_m_ of ~3.0 µM) towards free and immobilized Co-based NFs was described [40], whereas a 1.5-fold decline in substrate affinity was reported for *Tv*Lac and Zn-based NFs for an over-free laccase value of 6.3 µM [39]. After immobilization, the *V*_max_ was enhanced to 542 µmol∙min^−1^∙mg protein^−1^ for Fe_3_O_4_/Mn-*Tv*Lac, and 570 µmol∙min^−1^∙mg protein^−1^ for Mn-*Tv*Lac. In contrast, no considerable *K*_m_ and *V*_max_ variations were reported on *Tv*Lac towards ABTS after immobilization as Co-based NFs [42].

### 3.4. Storage, Thermal Stability, Reusability, and Leaching

**Figure 4:**
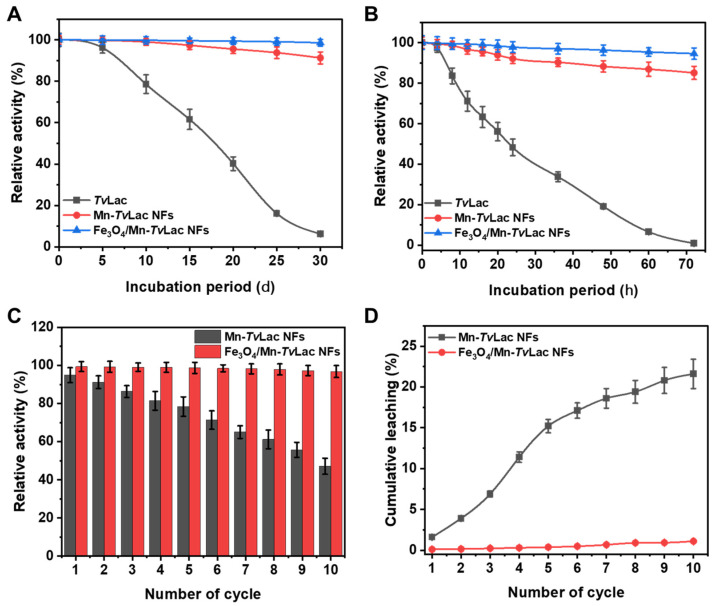
Storage stability of free and immobilized *Tv*Lac at (**A**) room temperature, (**B**) 4 °C, (**C**) reusability, and (**D**) leaching.

Enzyme properties after immobilization, especially positive alternation in activity for better stability, are essential for defining the success of immobilization methods [39,47,48]. Initially, the room temperature storage stability of free *Tv*Lac, Fe_3_O_4_/Mn-*Tv*Lac, and Mn-*Tv*Lac NFs were compared after incubation up to 72 h under optimum pH conditions at 25 °C (Figure 4A). A successive decline in the activity of free *Tv*Lac was observed during the increasing incubation period, with ~99% loss of residual activity at 72 h. Under similar incubation conditions, Mn-*Tv*Lac and Fe_3_O_4_/Mn-*Tv*Lac NFs retained significantly higher residual activities of 85.1% and 94.6%, respectively. Here, the beneficial influence of immobilization was observed via improvements in *Tv*Lac’s storage stability by up to 135-fold. Previously, Cu-based immobilized laccase NFs showed a minor 4-fold improvement over that of free enzymes upon storage at room temperature [48]. Following one month of storage at 4 °C, the residual activity of free *Tv*Lac declined to 6.3% (Figure 4B). At similar incubation periods, Mn-*Tv*Lac and Fe_3_O_4_/Mn-*Tv*Lac NFs lost only marginal residual activities of 8.7% and 1.4%, respectively. Previously, Co-based laccase NFs demonstrated an insignificant 1.4-fold improvement, corresponding to free enzymes, after 40 days of incubation [39]. Meanwhile, Cu-derived laccase NFs at 4 °C storage for 10 days exhibited analogous residual activity [48]. Furthermore, the thermal stability of free *Tv*Lac, immobilized Fe_3_O_4_/Mn-*Tv*Lac, and Mn-*Tv*Lac NFs were investigated under optimum pH conditions at various temperatures to validate successful immobilization (Table 3). Thermostability measured for free *Tv*Lac at different temperatures showed a *t*_1/2_ value of 12.2 h (40 °C), 9.12 h (45 °C), 6.21 h (50 °C), 4.03 h (60 °C), and 0.90 h (70 °C). Whereas, Mn-*Tv*Lac exhibited a *t*_1/2_ of 25.6, 18.3, 14.1, 11.9, and 3.80 h at 40, 45, 50, 60, and 70 °C under similar conditions, respectively. In contrast, Fe_3_O_4_/Mn-*Tv*Lac showed higher stability, with up to 2.5-, 2.9-, 3.4-, 4.3-, and 8.6-fold higher *t*_1/2_ values at the corresponding temperatures than those of free *Tv*Lac. Previously, at 45 °C, laccase immobilized as Co-based NFs exhibited reduced residual activity compared to the free enzyme [39].

After successive recycling, the activities of Fe_3_O_4_/Mn-*Tv*Lac and Mn-*Tv*Lac NFs were reduced (Figure 4C). For the Mn-*Tv*Lac NFs, a decline in residual activity to 78.4% and 47.1% was observed after five and 10 reuse cycles, respectively. Under similar recycling conditions, Fe_3_O_4_/Mn-*Tv*Lac retained much better residual activities of 98.7% and 96.8%, respectively. Under various recycling conditions, an immobilized system based on purified laccase exhibited residual activities of (i) ~50% after 10 cycles for Co-based, and dendrimer-grafted silica-coated hercynite-copper phosphate magnetic NFs [39,47], (ii) 25–40% after five cycles for ferrite microparticles based on Ni-Zn and Ni-Zn-Co [49], (iii) 30% after 10 cycles for Cu-based NFs [45], and (iv) 40% after 12 cycles for Zn-based NFs [41]. During the reusability test, successive decreases in the immobilized laccase residual activity of Mn-*Tv*Lac can be linked to the leaching of bound enzymes or their inactivation due to the deformation of the NF matrix [18,40]. Further, higher cumulative leaching is confirmed by higher structural deformation in Mn-*Tv*Lac than Fe_3_O_4_/Mn-*Tv*Lac (Figure S7). The high reuse stability of Fe_3_O_4_/Mn-*Tv*Lac was validated by its remarkably low cumulative leaching of 1.1% compared to the 21.6% of Mn-*Tv*Lac (Figure 4D).

### 3.5. Acute Toxicity Analysis of Nanoflowers

Nanomaterials, especially nanoparticles, are toxic to aquatic biodiversity [50]. The Fe_3_O_4_/Mn-*Tv*Lac exhibited an EC_50_ of 980 µg∙mL^−1^ compared to the 325 µg∙mL^−1^ of pure Fe_3_O_4_ nanoparticles for *V. fischeri* (Table 4). Whereas, synthesized NFs without Fe_3_O_4_ have considerably lower toxicity than Fe_3_O_4_/Mn-*Tv*Lac with an EC_50_ of 695 µg∙mL^−1^. Here, the larger size of Fe_3_O_4_/Mn-*Tv*Lac (7–9 μm) can be associated with a lower toxicity than Fe_3_O_4_ nanoparticles (~50 nm) that can quickly internalize within the cell via cell membrane pores (75–90 nm) and inhibit *V. fischeri* growth [51,52]. This finding implies that the synthesized magnetic NFs are more eco-friendly than pure Fe_3_O_4_ nanoparticles since they require ~3.0-fold higher concentrations to achieve a 50% viability reduction in *V. fischeri*. Previously, dust samples containing Fe and Mn showed high toxicity (90%) toward *V. fischeri* [51]. With a lower incubation period of 5 min, an EC_50_ of 240 µg∙mL^−1^ was reported for Fe_3_O_4_ nanoparticles against *V. fischeri* [52].

### 3.6. Decolorization of Synthetic Dyes in the Presence of Inhibitors via Immobilized TvLac

**Figure 5:**
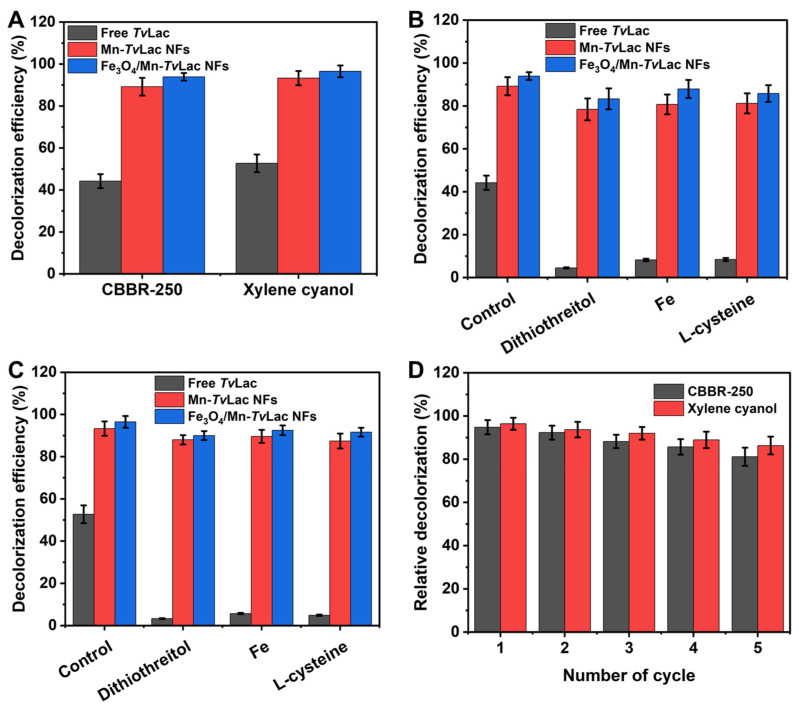
Decolorization of dyes by free and immobilized *Tv*Lac (**A**), decolorization in the presence of laccase inhibitors for CBBR-250 (**B**), and xylene cyanol (**C**), and reusability in the presence of 0.1 mM of Fe(II) (**D**).

To investigate the prospective application of free and immobilized *Tv*Lac as Mn-*Tv*Lac and Fe_3_O_4_/Mn-*Tv*Lac NFs, the decolorization of CBBR-250 and xylene cyanol was evaluated at 25 °C during a 48 h incubation period and under agitation (100 rpm). The decolorization of dyes was enhanced with an increase in the incubation period up to 48 h (Table S5). Subsequently, the decolorization efficiency stabilized after 72 h of incubation. Incomplete decolorization may be associated with enzyme inactivation or substrate inhibition. In contrast, Fe_3_O_4_ nanoparticles and heat-inactivated Fe_3_O_4_/Mn-*Tv*Lac, used as controls, showed less than 1% dye decolorization, which could be associated with their inherent properties for dye adsorption. Free *Tv*Lac showed decolorization efficiencies of 44.2% and 52.7% for CBBR-250 and xylene cyanol, respectively (Figure 5A). Under similar incubation conditions, the Mn-*Tv*Lac NFs exhibited higher decolorization efficiencies (89.2% for CBBR-250 and 93.3% for xylene cyanol), whereas a maximum decolorization of 93.9% and 96.5% for CBBR-250 and xylene cyanol was observed by Fe_3_O_4_/Mn-*Tv*Lac NFs, respectively. Previously, lower decolorization efficiencies were reported in the range of 5–40% for Coomassie Brilliant Blue by *Aspergillus oryzae*-, *Paraconiothyrium varia-bile*-*,* and *T. versicolor*-based laccases [53]. In contrast, a low 36% decolorization of Coomassie Brilliant Blue by immobilized laccase from *Myceliophthora thermophila* on epoxy-functionalized silica was observed over free laccase with a value of 53% under similar reaction conditions [54]. Xylene cyanol decolorization of up to 35% was demonstrated using *Phanerochaete chrysosporium*-based lignin peroxidase combined with glucose oxidase in the presence of external hydrogen peroxide [55]. Similar dyes such as Brilliant Blue X-BR and Remazol Brilliant Blue R have been used for free and immobilized *Tv*Lac on chitosan beads [56]. Dithiothreitol, Fe(II) ions, and l-cysteine are well-known potent inhibitors of laccase that can potentially inhibit laccase bioremediation during waste treatment [36,57]. Therefore, decolorization of these dyes was assessed in the presence of these inhibitors. A remarkable decline in the decolorization efficiency from 44.2% to 4.5% for CBBR-250 and from 52.7% to 3.3% for xylene cyanol by *Tv*Lac was observed in the presence of these potent laccase inhibitors (Figure 5B,C). In contrast, Mn-*Tv*Lac and Fe_3_O_4_/Mn-*Tv*Lac retained excellent residual decolorization efficiencies of up to 87.9% for CBBR-250 and up to 92.5% for xylene cyanol. Furthermore, repeated batch decolorization of these dyes occurred when using Mn-*Tv*Lac and Fe_3_O_4_/Mn-*Tv*Lac NFs in the presence of 0.1 mM Fe(II). After five reuse cycles, Mn-*Tv*Lac and Fe_3_O_4_/Mn-*Tv*Lac retained residual decolorization efficiencies of up to 81.1% and 86.3% for CBBR-250 and xylene cyanol, respectively (Figure 5D). Previously, a low Brilliant Blue decolorization of 63.2% was reported for *Tv*Lac immobilized on lectin-modified cryogels [58]. These findings suggest that the immobilization of *Tv*Lac using magnetic nanoparticles and Mn metals as inorganic protein hybrid NFs is beneficial for retaining a high decolorization efficiency in the presence of enzyme inhibitors.

## 4. Conclusions

Enzyme immobilization plays a crucial role in various biotechnological applications, including in biocatalysis and wastewater treatment. In this study, a green synthesis approach was successfully employed to synthesize magnetic inorganic-protein hybrid NFs using partially purified *Tv*Lac, Fe_3_O_4_ nanoparticles, and Mn for potential application in the decolorization of dyes in the presence of inhibitors. The synthesized Fe_3_O_4_/Mn-*Tv*Lac and Mn-*Tv*Lac NFs showed broad pH and temperature profiles, improved catalytic properties, and high storage and thermostability compared to those of free *Tv*Lac. Incorporating Fe_3_O_4_ nanoparticles was found to be more suitable for achieving higher reusability and decolorization efficiency of dyes in the presence of known potent laccase inhibitors than when free enzymes are used. The synthesized NFs exhibited superior biocompatibility towards *V. fischeri* when compared to the Fe_3_O_4_ nanoparticles. This is the first report demonstrating the *Tv*Lac- and Mn-based one-pot synthesis of eco-friendly magnetic protein-inorganic hybrids that can be utilized for broad biotechnological applications.

## Figures and Tables

**Table 1:** Immobilization of *Tv*Lac as inorganic-protein hybrid nanobiocatalysts as NFs.

Hybrids	Encapsulation Yield (%)	Relative Activity (%) ^a^
Mn-*Tv*Lac	90.3 ± 2.2	260 ± 18.4
Fe_3_O_4_/Mn-*Tv*Lac	85.5 ± 3.6	245 ± 14.7

^a^ The free *Tv*Lac activity of 212 U mg of total protein^−1^ was considered as 100%.

**Table 2:** Kinetic parameters of free and immobilized partially purified *Tv*Lac as inorganic-protein hybrid NFs.

Enzyme	*V*_max_ (µmol min^−1^ mg Protein^−1^)	*K*_m_ (µM)
Free *Tv*Lac	219 ± 14.6	38.1 ± 2.4
Mn-*Tv*Lac NFs	570 ± 37.8	17.1 ± 1.2
Fe_3_O_4_/Mn-*Tv*Lac NFs	542 ± 33.2	17.6 ± 1.3

**Table 3:** The thermal deactivation constant (*k*_d_) and *t*_1/2_ of free and immobilized *Tv*Lac as inorganic-protein hybrid NFs.

Temperature (°C)	Free *Tv*Lac		Mn-*Tv*Lac NFs		Fe_3_O_4_/Mn-*Tv*Lac NFs	
	*k*_d_ (h^−1^)	*t*_1/2_ (h)	*k*_d_ (h^−1^)	*t*_1/2_ (h)	*k*_d_ (h^−1^)	*t*_1/2_ (h)
40	0.057 ± 0.005	12.2 ± 0.9	0.027 ± 0.002	25.6 ± 1.6	0.023 ± 0.002	30.5 ± 2.4
45	0.076 ± 0.006	9.12 ± 0.6	0.038 ± 0.003	18.3 ± 1.4	0.026 ± 0.002	26.4 ± 1.9
50	0.112 ± 0.009	6.21 ± 0.3	0.049 ± 0.004	14.1 ± 1.1	0.033 ± 0.003	21.1 ± 1.7
60	0.173 ± 0.014	4.03 ± 0.2	0.058 ± 0.005	11.9 ± 0.9	0.040 ± 0.003	17.2 ± 1.2
70	0.770 ± 0.051	0.90 ± 0.1	0.182 ± 0.015	3.80 ± 0.3	0.090 ± 0.007	7.71 ± 0.6

**Table 4:** The acute toxicity level of Fe_3_O_4_ nanoparticles and synthesized inorganic-protein hybrid NFs.

Particles	EC_50_^a^ Concentration (µg mL^−1^)
Fe_3_O_4_ nanoparticles	325 ± 30
Mn-*Tv*Lac NFs	695 ± 55
Fe_3_O_4_/Mn-*Tv*Lac NFs	980 ± 73

^a^ EC_50_ was determined using the bioluminescence of *V. fischeri* after 30 min of incubation.

## Data Availability

Data are contained within the article.

## Supplementary Materials

The following supporting information can be downloaded at: https://www.mdpi.com/article/10.3390/ma17081790/s1, Table S1. Details of the partial purification of *Trametes versicolor* laccase; Table S2. Activity of partially purified *Trametes versicolor* laccase in the presence of manganese ions; Table S3: Immobilization of *Tv*Lac as magnetic inorganic-protein hybrid nanobiocatalysts as NFs at different incubation; Table S4: The activity measurements of *Tv*Lac using various substrates; Table S5: The decolorization of dyes by free and immobilized laccase as NFs; Figure S1. *Trametes versicolor* laccase production profile in the presence of different inducers (0.5 mM). Figure S2. Activity measurements by the spectrophotometric procedure for produced laccase; Figure S3: The field-emission scanning electron microscopy images of magnetic nanoflowers synthesized after 3 h (A), 12 h (B), and 24 h (C) incubation at 4°C; Figure S4: Field-emission scanning electron microscopy and elemental mapping analysis of Mn_3_(PO_4_)_2_-laccase (Mn-*Tv*Lac) nanoflowers. (A) field-emission scanning electron micrograph of the inorganic-protein hybrids as Mn-*Tv*Lac nanoflowers, and (B-E) elemental mapping analysis for nitrogen (B), oxygen (C), manganese (D), and phosphorus (E); Figure S5: X-ray diffraction patterns of inorganic-protein hybrid nanoflowers (NFs). Mn-*Tv*Lac = Mn_3_(PO_4_)_2_-laccase; Figure S6: Magnetic property measurements of Fe_3_O_4_ nanoparticles and Fe_3_O_4_/Mn-*Tv*Lac hybrids (A), recovery of magnetic NFs by applying an external magnetic field (B), and under recycling conditions (C); Figure S7: The field-emission scanning electron microscopy images of Fe_3_O_4_/Mn-*Tv*Lac (A and B) and Mn-*Tv*Lac NFs (C and D): before recycling (A and C) and after ten cycles of reusability (B and D).
