# Supplementary material for: Trametes versicolor Laccase-Based Magnetic Inorganic-Protein Hybrid Nanobiocatalyst for Efficient Decolorization of Dyes in the Presence of Inhibitors
Source: Materials (Basel). 2024 Apr 13;17(8):1790. doi: 10.3390/ma17081790 (PMC11051536; doi:10.3390/ma17081790)
Supplement: Supplementary file 1 [file materials-17-01790-s001.zip › materials-2910151-supplementary.pdf]

materials

Supplementary Materials

Trametes versicolor laccase-based magnetic inorganic-protein hybrid nanobiocatalyst for efficient decolorization of dyes in presence of inhibitors

Sanjay K. S. Patel [1,+], Rahul K. Gupta [1,+], Karthikeyan K. Karuppanan [1], Deepak K. Padhi [1], Sampathkumar Ranganathan [1], Parasuraman Paramanantham [1] and Jung-Kul Lee [1,*]

Department of Chemical Engineering, Konkuk University, Seoul 05029, Republic of Korea; sanjaykspatel@gmail.com (S.K.S.P.); guptarahul9m@gmail.com (R.K.G.); karthikk1529@gmail.com (K.K.K.); dkpadhi7@gmail.com (D.K.P.); sampathbiotech@gmail.com (S.R.); parasupondiuni@gmail.com (P.P.)

* Correspondence: jkrhee@konkuk.ac.kr

† These authors equally contributed to this work.

materials MDPI

Table S1. Details of the partial purification of *Trametes versicolor* laccase.

Purification step	Volume (mL)	Total protein (mg)	Specific activity (U·mg total protein^{-1})	Yield (%)	Fold purification
Crude	100	2.1	160	100	1
30 kD purification	4.8	1.4	212	66.6	1.5

Table S2. Activity of partially purified *Trametes versicolor* laccase in the presence of manganese ions.

Metal ions concentration (mM)	Relative activity (%)
0	100 ± 3.2
0.8	108 ± 3.8
1.0	112 ± 5.6
2.0	127 ± 8.9
5.0	103 ± 4.5

[a]Free *Trametes versicolor* activity of 212 U·mg total protein^{-1} was considered 100%.

Table S3. Immobilization of *Tv*Lac as magnetic inorganic-protein hybrid nanobiocatalysts as NFs at different incubation.

Incubation (h)	Encapsulation yield (%)	Relative activity (%)[a]
2	46.1 ± 3.4	160 ± 13.0
6	72.9 ± 4.5	193 ± 15.2
24	85.5 ± 3.6	245 ± 14.7

[a]The free *Tv*Lac activity of 212 U mg of total protein^{-1} was considered as 100%.

Table S4. The activity measurements of *Tv*Lac using various substrates.

Substrate	Absorbance	Molar extinction coefficient (ε_{max}·M^{-1}·cm^{-1})	Relative activity (%)[a]
ABTS	420	36000	100 ± 4.3
2,6-dimethoxy phenol	470	35645	51.7 ± 3.7
Guaiacol	436	6400	39.2 ± 2.8
Pyrogallol	450	4400	16.8 ± 1.2

[a]The activity was measured using 1.0 mM substrate under standard assay conditions. Free *Tv*Lac activity of 212 U mg of total protein^{-1} was considered 100%.

materials

MDPI

Table S5. The decolorization of dyes by free and immobilized laccase as NFs.

System	Decolorization (%)				
	6 h	12 h	24 h	48 h	72 h
			CBBR-250		
Free *Tv*Lac	16.3 ± 1.2	27.6 ± 2.1	40.1 ± 2.6	44.2 ± 2.7	44.3 ± 2.7
Mn-*Tv*Lac NFs	27.8 ± 1.8	53.9 ± 3.8	85.6 ± 3.7	89.2 ± 3.1	89.4 ± 3.2
Fe_3O_4/Mn-*Tv*Lac NFs	27.5 ± 2.0	56.3 ± 4.2	89.5 ± 3.3	93.9 ± 1.9	94.3 ± 1.8
Fe_3O_4 nanoparticles	0.22 ± 0.1	0.22 ± 0.1	0.22 ± 0.1	0.22 ± 0.1	0.22 ± 0.1
Fe_3O_4/Mn-*Tv*Lac NFs (heat-inactivated)[a]	0.56 ± 0.1	0.58 ± 0.1	0.59 ± 0.1	0.61 ± 0.1	0.62 ± 0.1
			Xylene cyanol		
Free *Tv*Lac	18.3 ± 1.4	31.8 ± 2.5	49.3 ± 2.9	52.7 ± 3.8	52.8 ± 3.9
Mn-*Tv*Lac NFs	31.0 ± 2.3	58.6 ± 4.4	89.4 ± 3.2	93.3 ± 2.4	93.5 ± 2.3
Fe_3O_4/Mn-*Tv*Lac NFs	30.4 ± 2.4	61.2 ± 4.7	92.7 ± 2.3	96.5 ± 1.6	96.9 ± 1.7
Fe_3O_4 nanoparticles	0.21 ± 0.1	0.22 ± 0.1	0.22 ± 0.1	0.22 ± 0.1	0.22 ± 0.1
Fe_3O_4/Mn-*Tv*Lac NFs (heat-inactivated)	0.64 ± 0.1	0.67 ± 0.1	0.69 ± 0.1	0.70 ± 0.1	0.70 ± 0.1

[a]Heat-inactivation of the enzyme was done at 90 °C for 4 h.

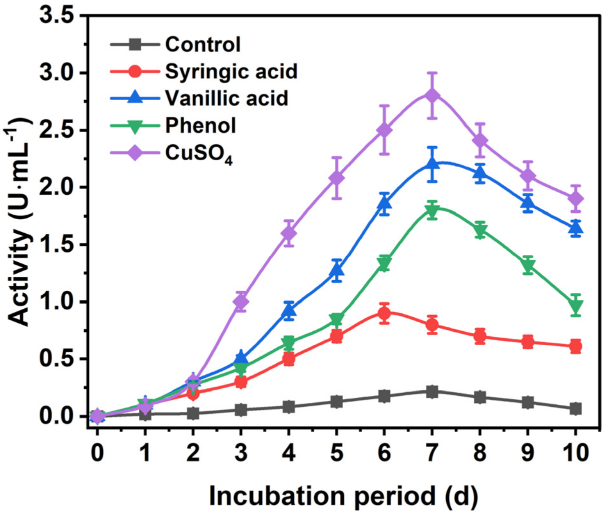

Figure S1. *Trametes versicolor* laccase production profile in the presence of different inducers (0.5 mM).

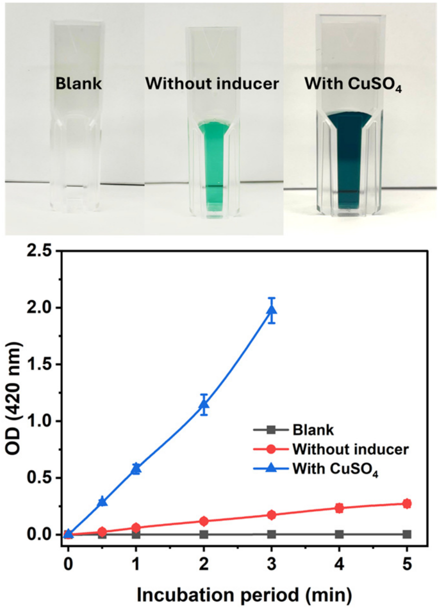

Figure S2. Activity measurements by the spectrophotometric procedure for produced laccase.

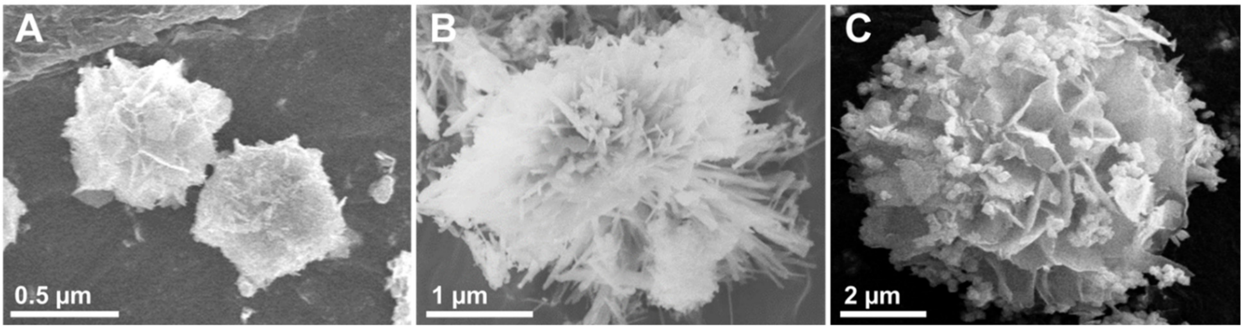

Figure S3. The field-emission scanning electron microscopy images of magnetic nanoflowers synthesized after 3 h (A), 12 h (B), and 24 h (C) incubation at 4°C.

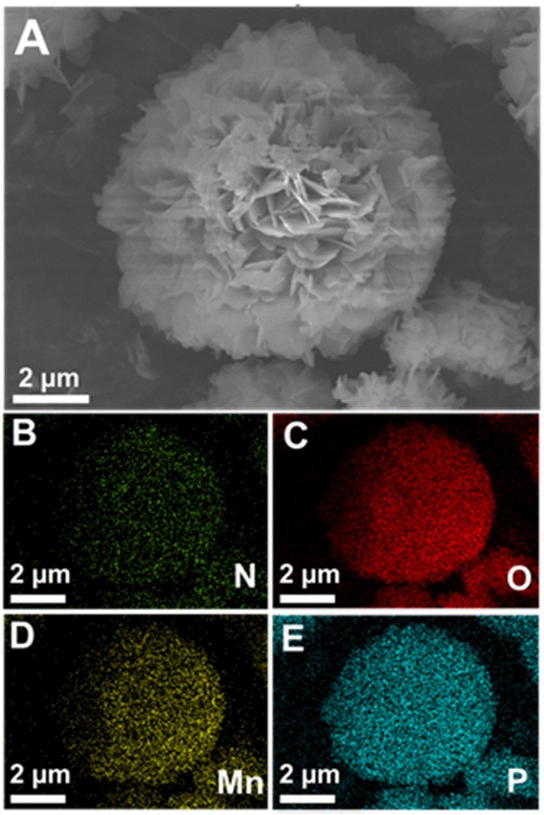

Figure S4. Field-emission scanning electron microscopy and elemental mapping analysis of $Mn_3(PO_4)_2$-laccase (Mn-*Tv*Lac) nanoflowers. (A) field-emission scanning electron micrograph of the inorganic-protein hybrids as Mn-*Tv*Lac nanoflowers, and (B-E) elemental mapping analysis for nitrogen (B), oxygen (C), manganese (D), and phosphorus (E).

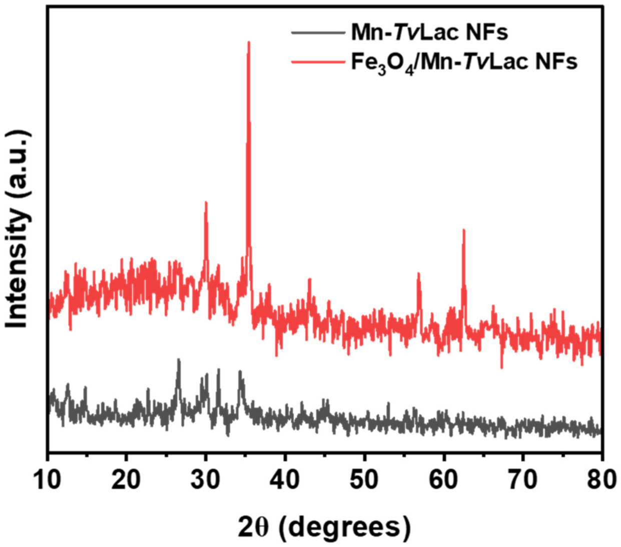

Figure S5. X-ray diffraction patterns of inorganic-protein hybrid nanoflowers (NFs). Mn-*Tv*Lac = $Mn_3(PO_4)_2$-laccase.

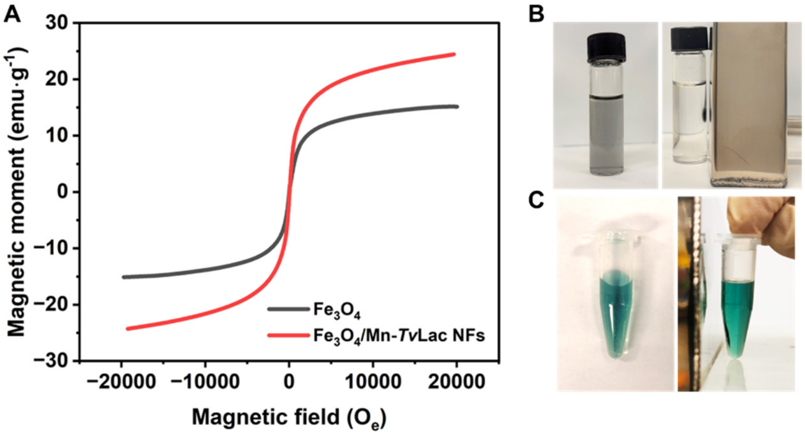

Figure S6. Magnetic property measurements of Fe_3O_4 nanoparticles and Fe_3O_4/Mn-*Tv*Lac hybrids (A), recovery of magnetic NFs by applying an external magnetic field (B), and under recycling conditions (C).

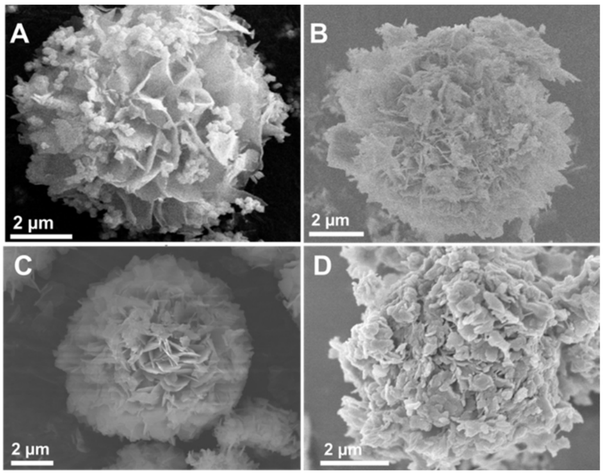

Figure S7. The field-emission scanning electron microscopy images of Fe_3O_4/Mn-*Tv*Lac (A and B) and Mn-*Tv*Lac NFs (C and D): before recycling (A and C) and after ten cycles of reusability (B and D).
